# Supplementary material for: An Anti-PSMA Immunotoxin Reduces Mcl-1 and Bcl2A1 and Specifically Induces in Combination with the BAD-Like BH3 Mimetic ABT-737 Apoptosis in Prostate Cancer Cells
Source: Cancers (Basel). 2020 Jun 22;12(6):1648. doi: 10.3390/cancers12061648 (PMC7352695; doi:10.3390/cancers12061648)
Supplement: Supplementary file 1 [file cancers-12-01648-s001.pdf]

Supplementary Figure S1

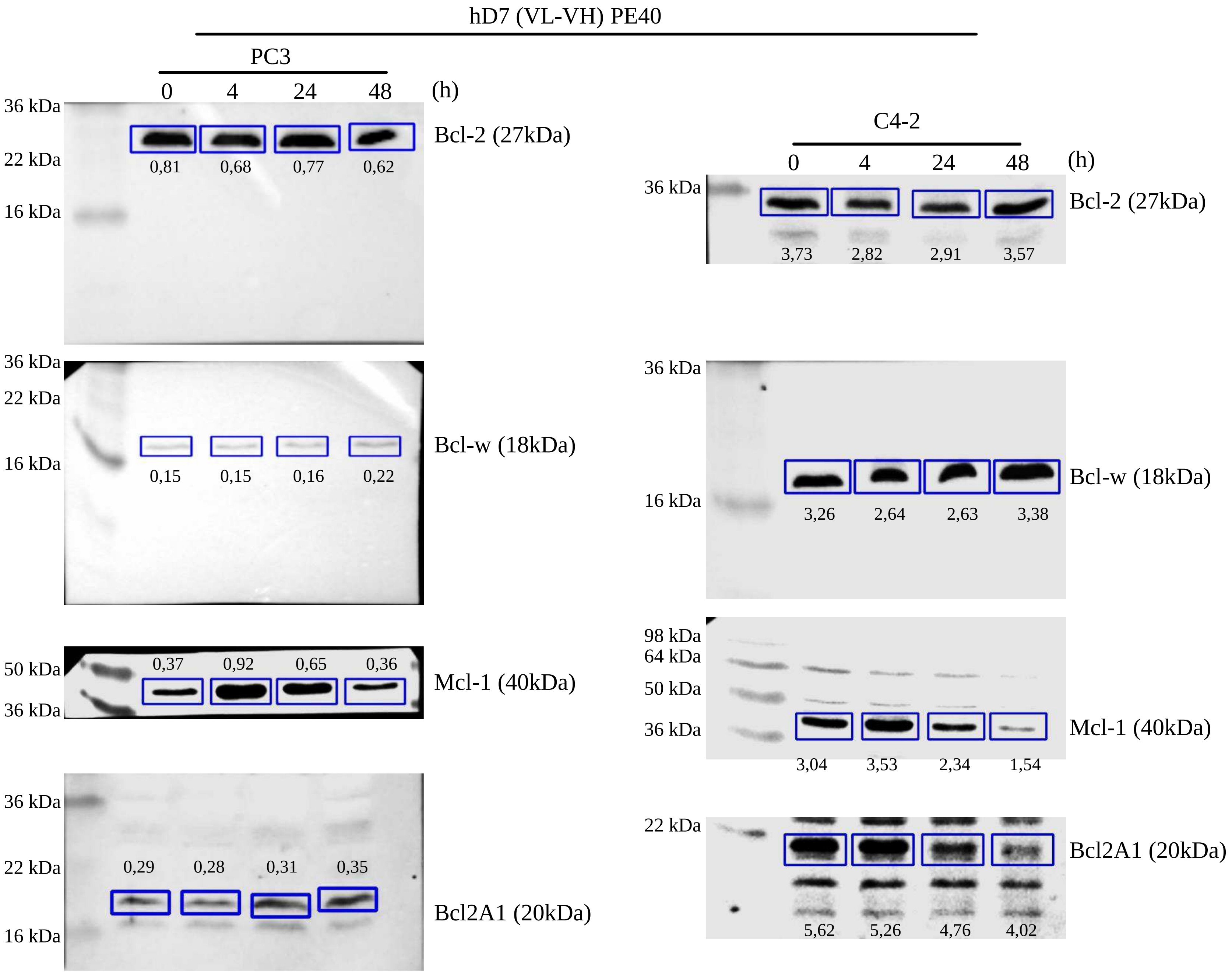

Supplementary Figure S1

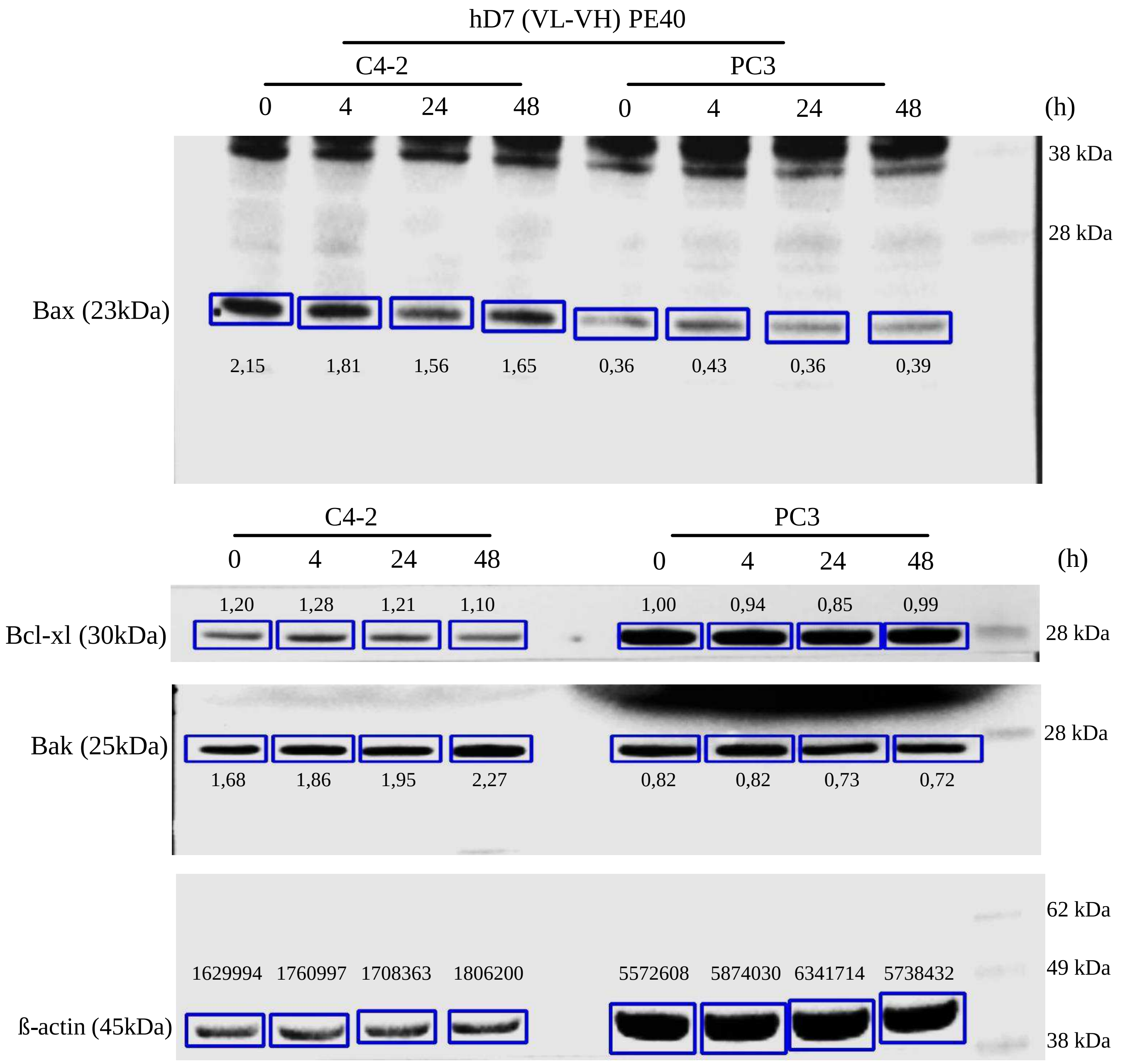

Supplementary Figure S1

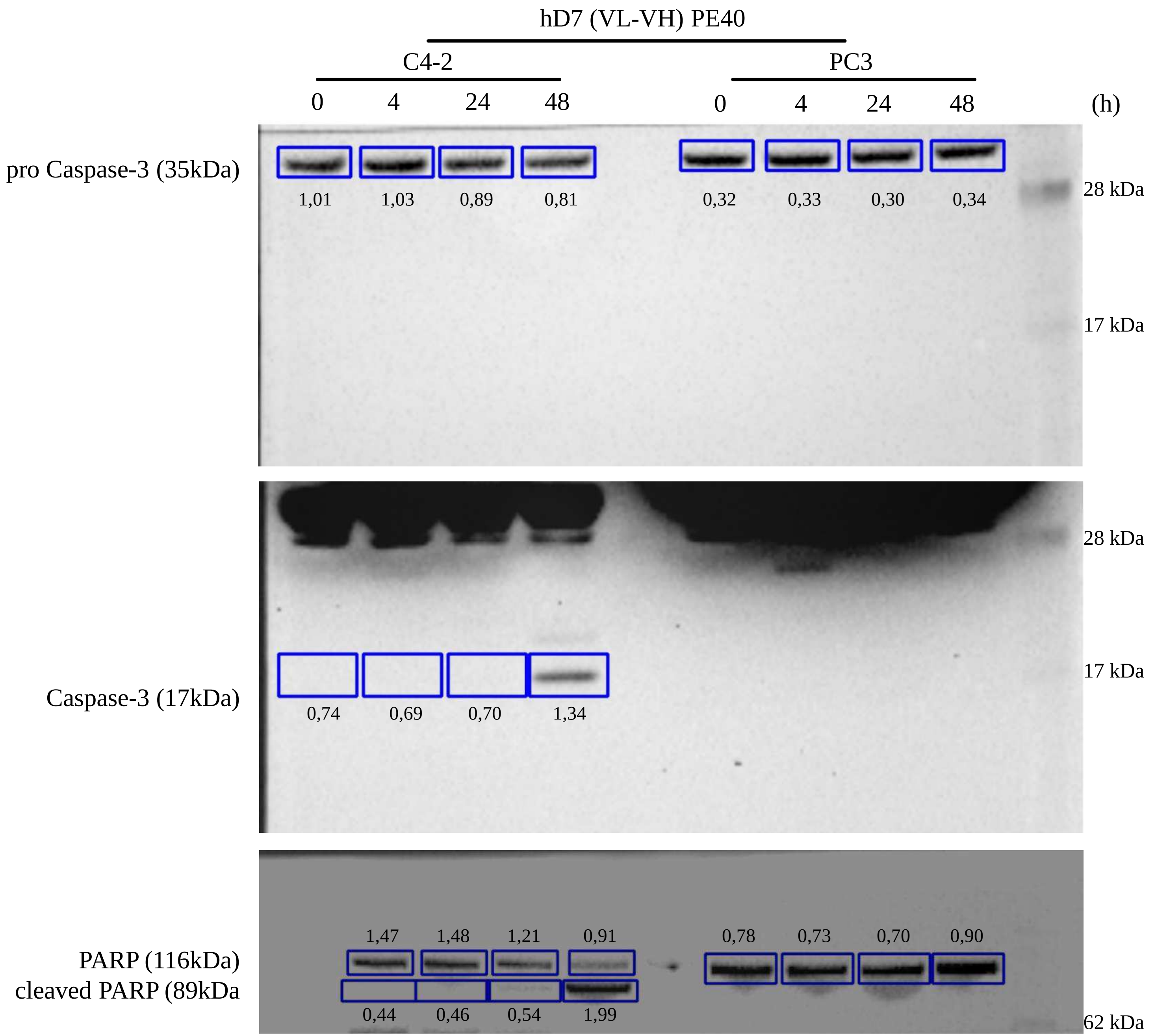

Supplementary Figure S1

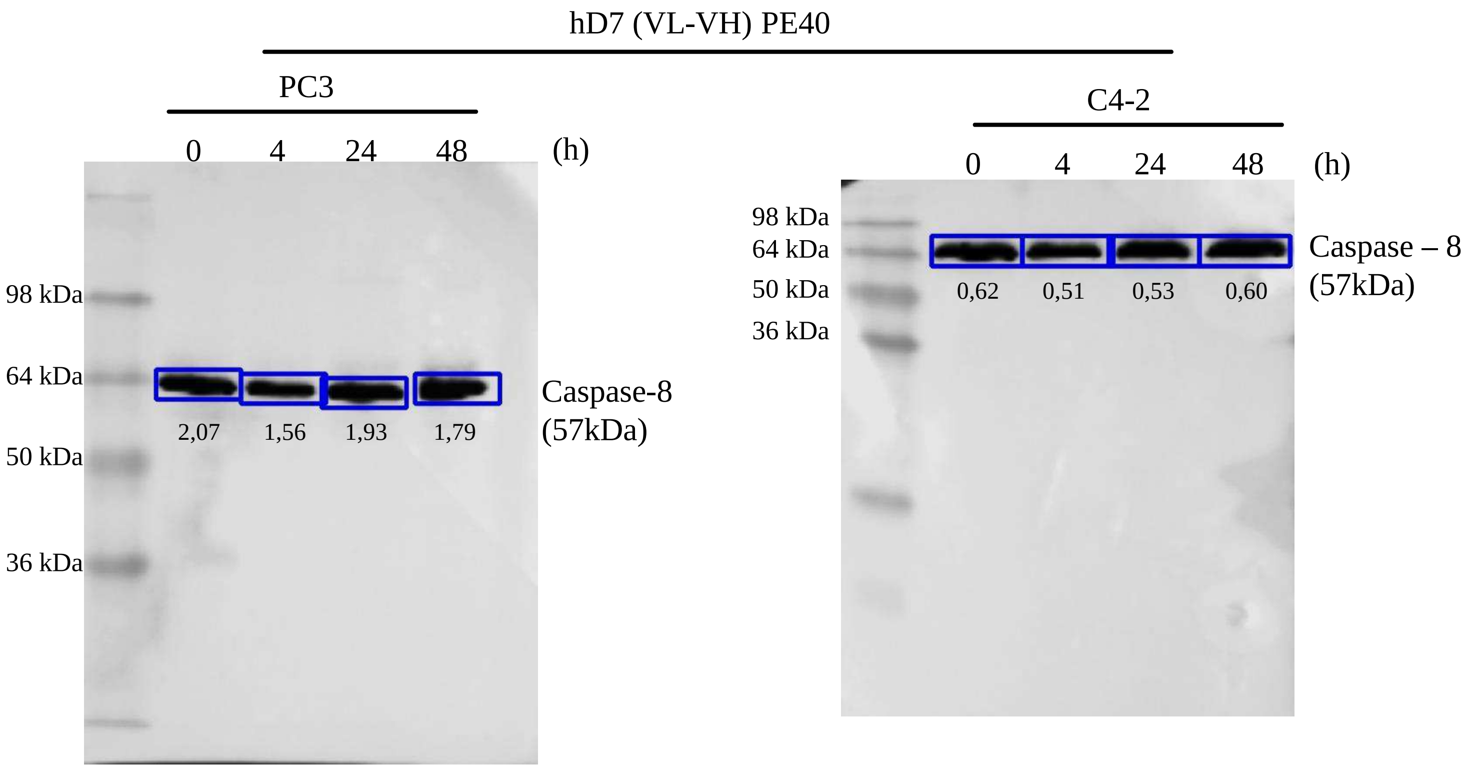

Supplementary Figure S1

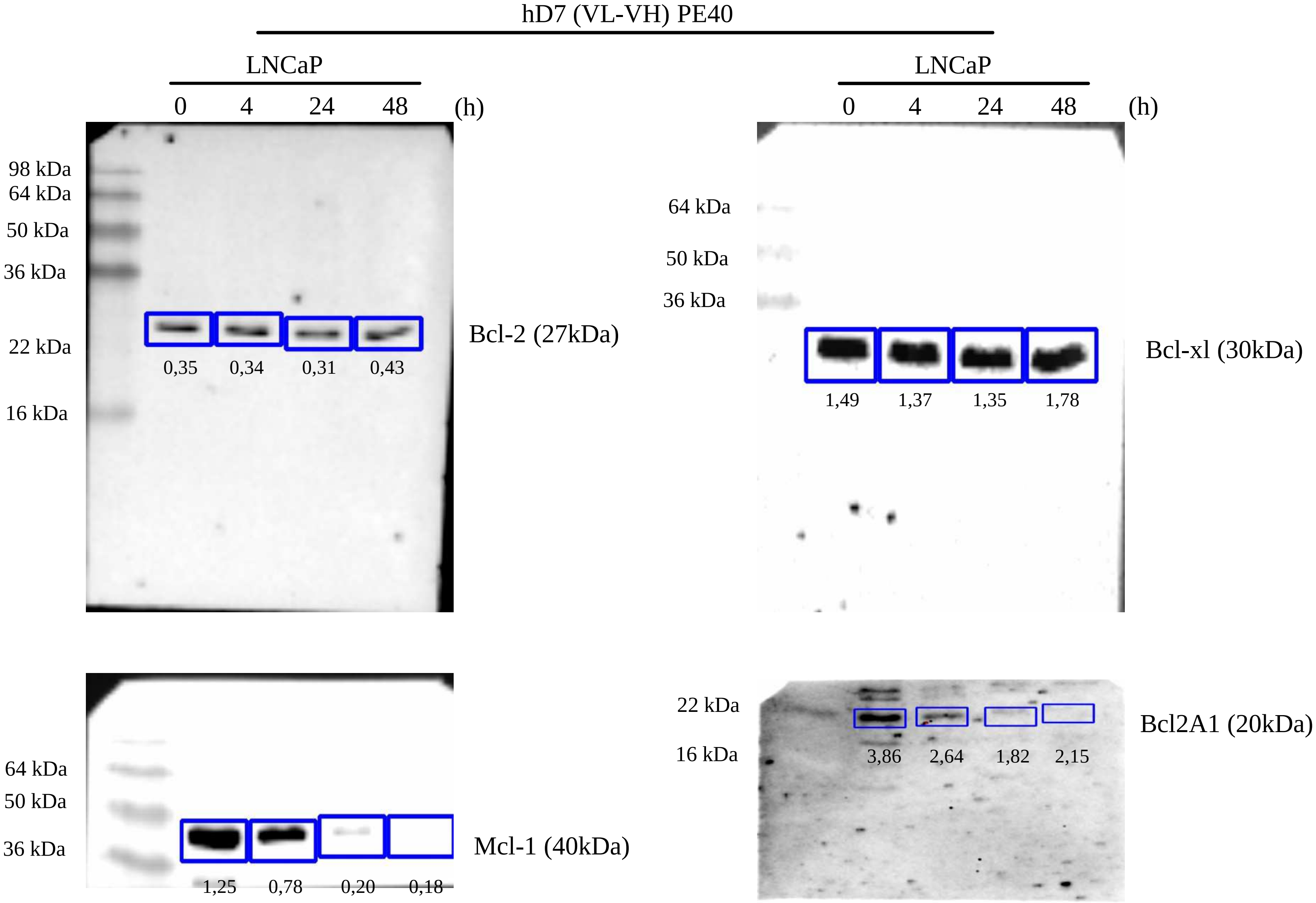

Supplementary Figure S1

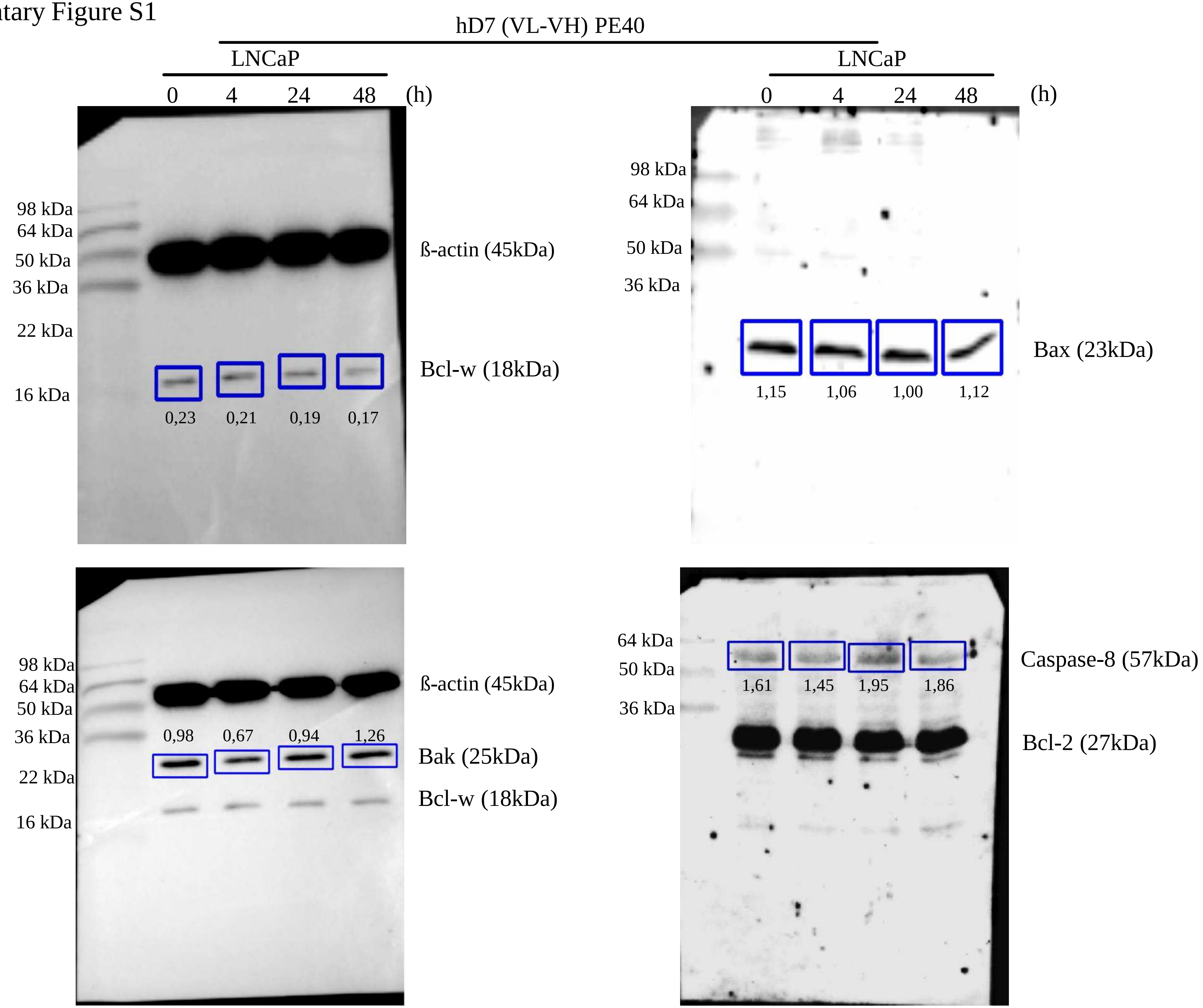

Supplementary Figure S1

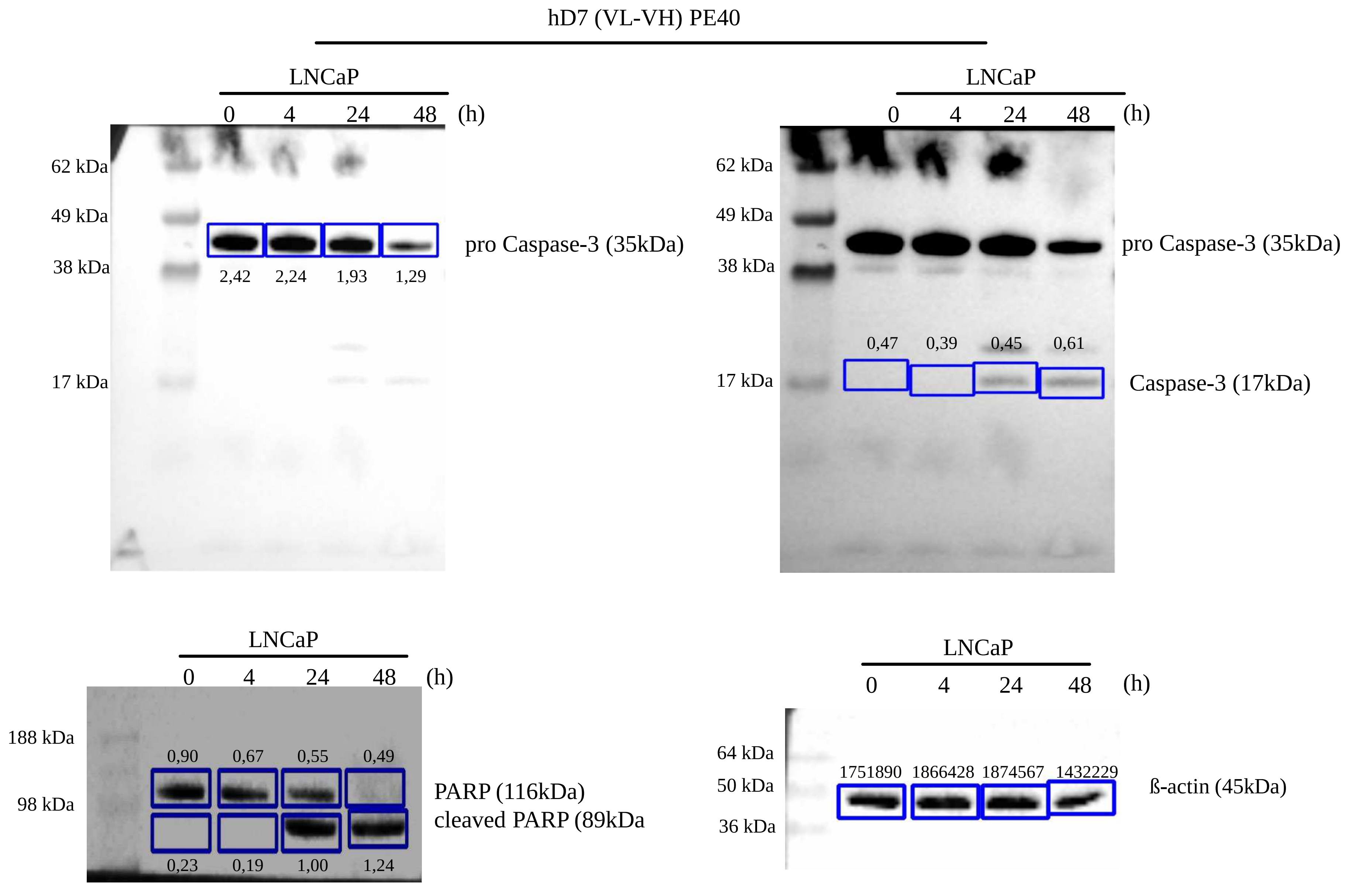

Supplementary Figure S1

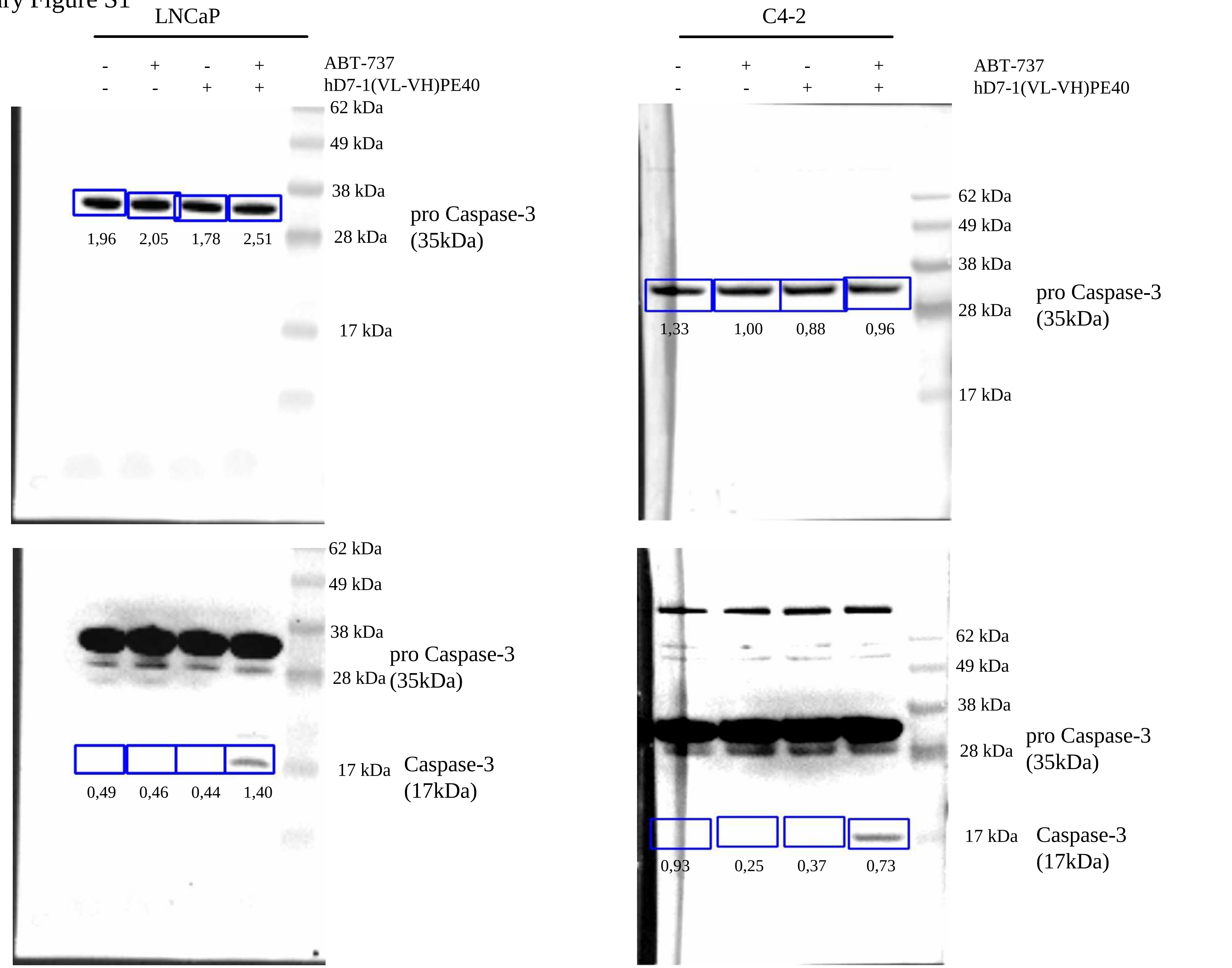

Supplementary Figure S1

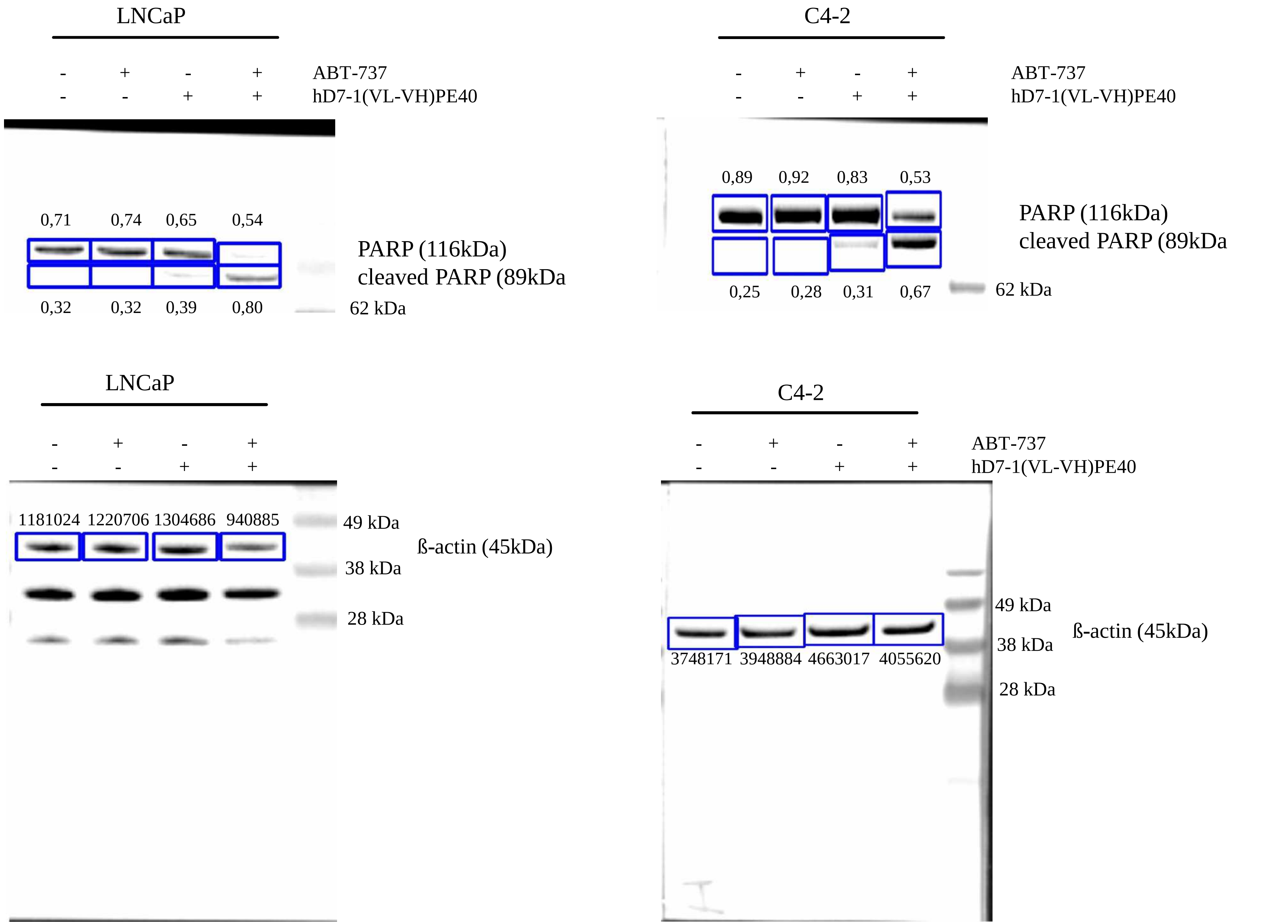

Supplementary Figure S1

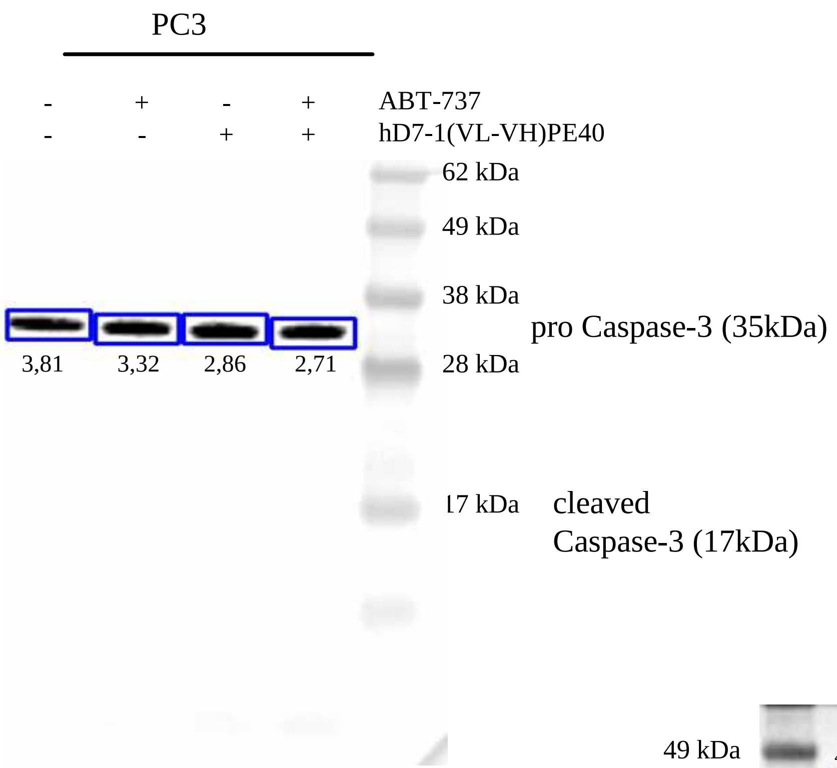

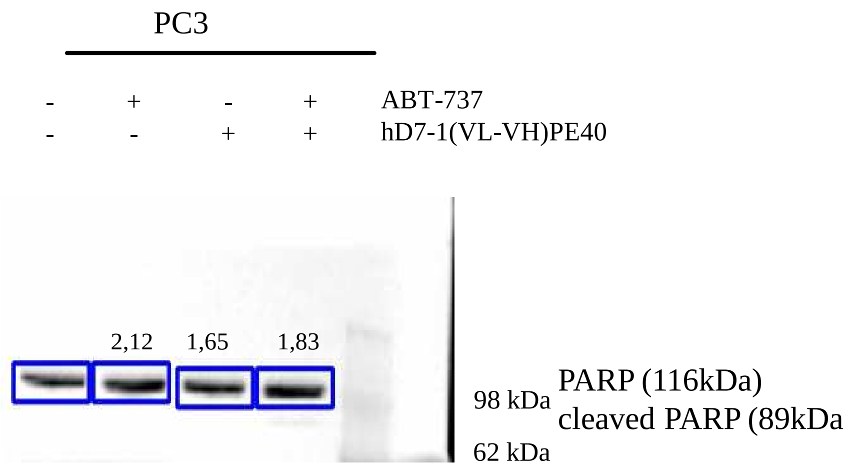

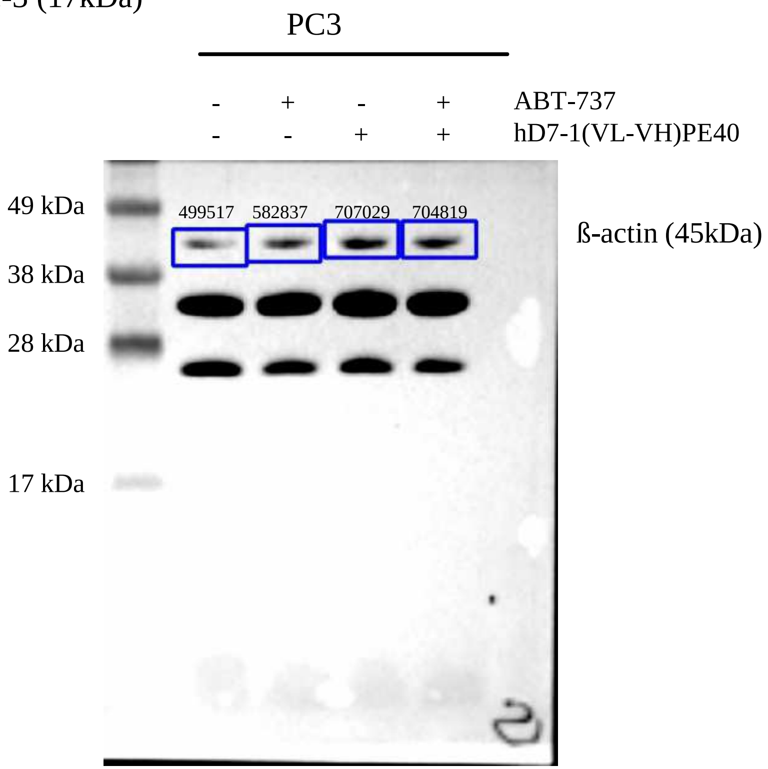

Supplementary Figure S1: Whole Western Blots including densitometry ratios.
Values corresponding to the volume of bands normalised to β-actin.

Supplementary Figure S2

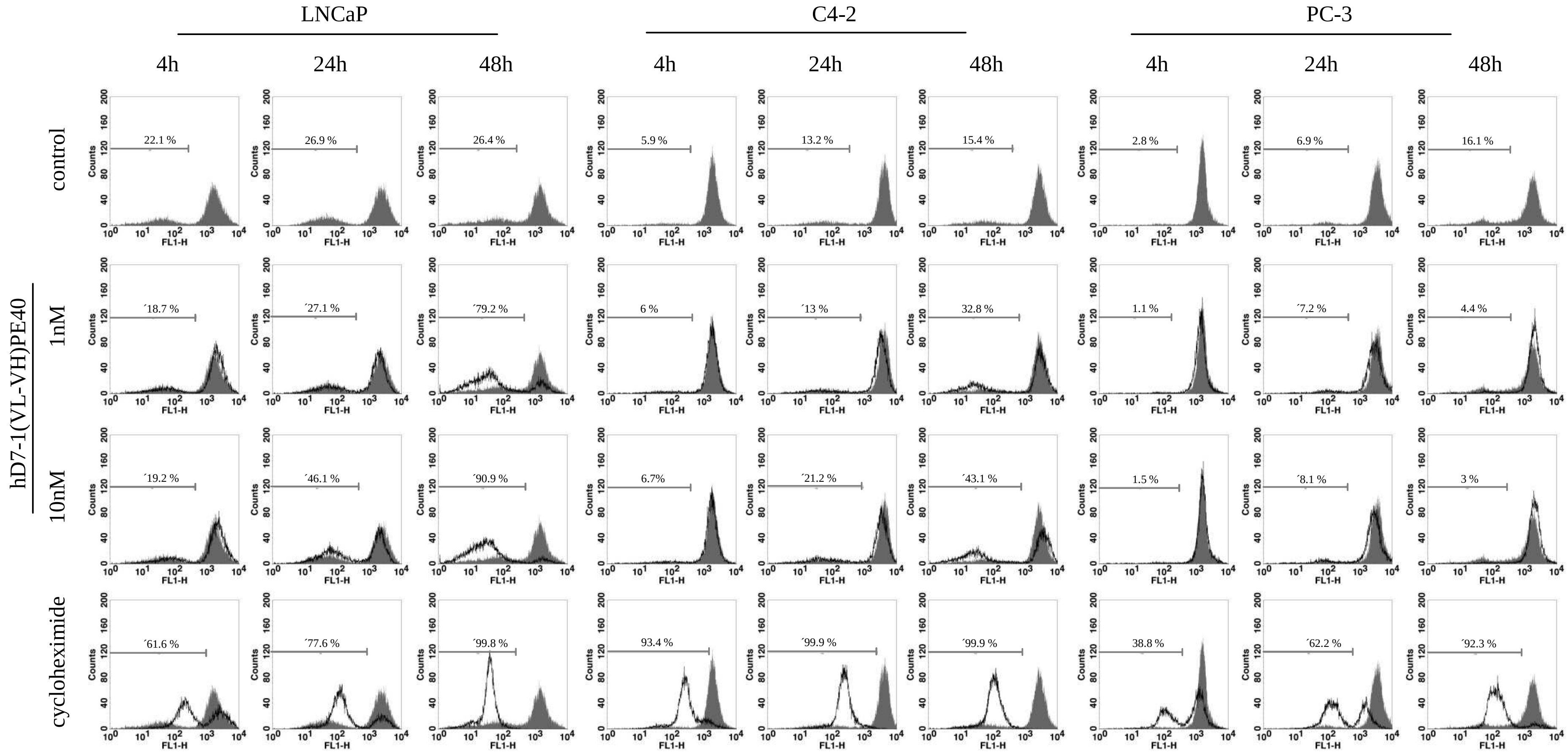

Supplementary Figure S2: The immunotoxin hD7-1(VL-VH)-PE40 induces MOMP in PSMA expressing LNCaP and C4-2 cells. Percentage of MOMP-positive cells was determined with help of the Rho-123 assay. Cycloheximide was used as control.

Supplementary Figure S3

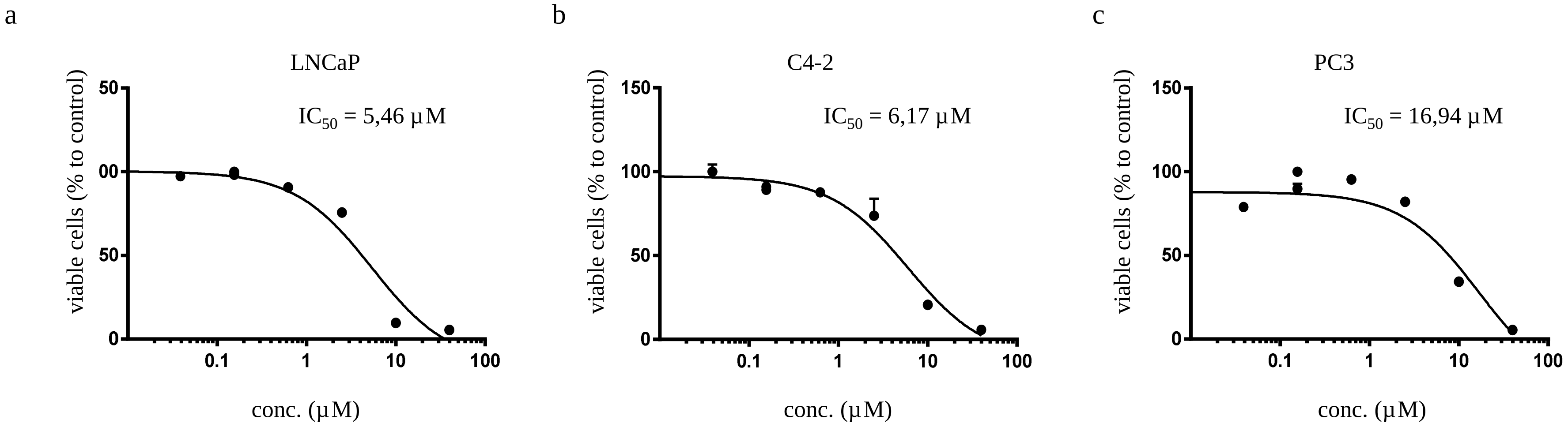

Supplementary Figure S3: Influence of ABT-737 on the viability of PC cells. (a) LNCaP (b) C4-2 and (c) PC3 cells were incubated with ABT-737. Cell viability was determined after 48 h by WST-1 test.

Supplementary Figure S4

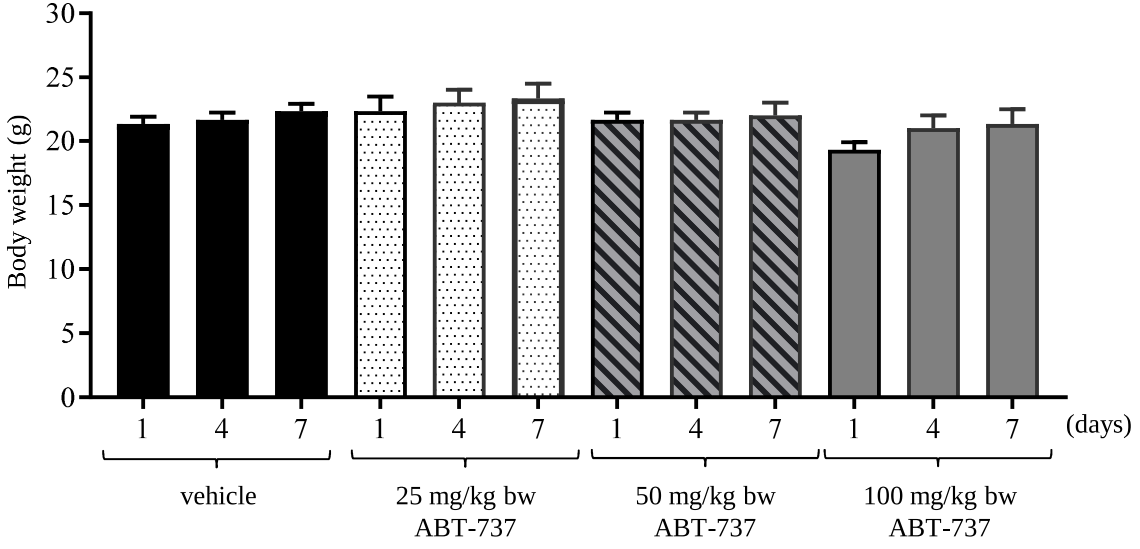

Supplementary Figure S4: Body weight analysis of mice treated with different doses of ABT-737 on day 1. Mean ± SD of three mice per group.

Supplementary Figure S5

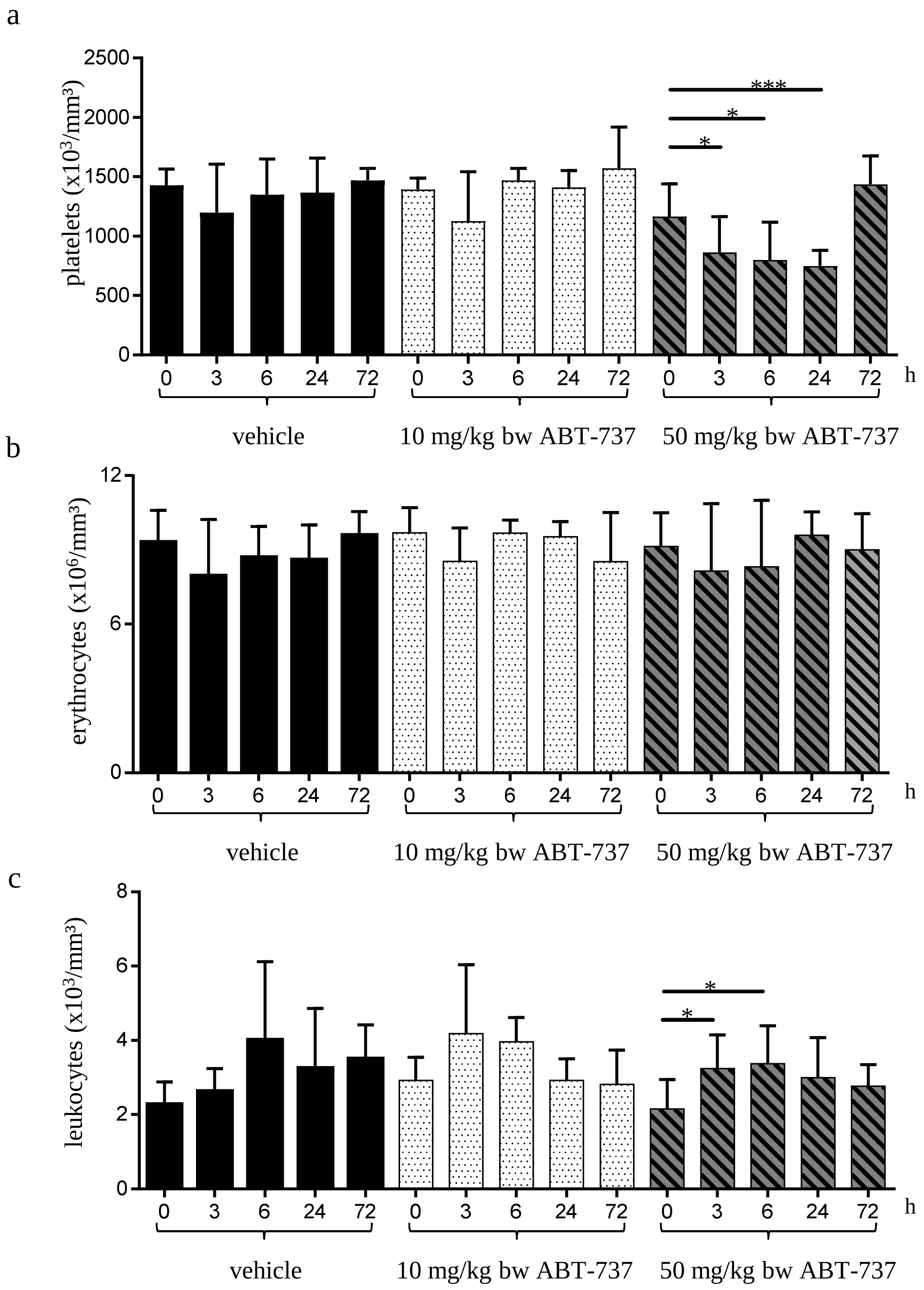

Supplementary Figure S5: ABT-737 induced transient thrombocytopenia in mice, which was accompanied by slight leukocytosis. SCID mice were treated with different doses of ABT-737. Control mice received the dilution buffer as vehicle. After different time points blood was drawn and blood cells were counted. Mean ± SD. Statistically significant differences were determined with unpaired t-test: *p<0.05, ***p<0.001.
